# Association between Estimated Cardiorespiratory Fitness and Depression among Middle-income Country Adults: Evidence from National Health Survey

**DOI:** 10.2174/1745017902117010198

**Published:** 2021-12-22

**Authors:** Eduardo Lattari, Andreza Jesus Costa Pascouto, Bruno Ribeiro Ramalho Oliveira, Livia Soares Silva, Aldair José Oliveira, Sérgio Machado, Geraldo Albuquerque Maranhao Neto

**Affiliations:** 1 Salgado de Oliveira University, Postgraduate Program in Physical Activity Sciences (PGCAF), Niterói, RJ, Brazil; 2 Rural Federal University of Rio de Janeiro, Physical Activity, Health, and Performance Research Laboratory, Department of Physical; Education and Sports, Seropédica, Brazil; 3 Rural Federal University of Rio de Janeiro, Laboratory of Social Dimensions Applied to Physical Activity and Sport (LABSAFE), Department of Physical; Education and Sports, Seropédica, Brazil; 4 Department of Sports Methods and Techniques, Federal University of Santa Maria, Santa Maria, Brazil; 5 Laboratory of Physical Activity Neuroscience, Neurodiversity Institute, Queimados- RJ, Brazil; 6 International Clinical Research Centre (ICRC), St Anne's University Hospital Brno (FNUSA), Brno-stred, Czech Republic.

**Keywords:** Fitness, Depression, Regression, Epidemiology, Cardiometabolic, Demographic

## Abstract

**Objective:**

This study assessed the relationship between cardiorespiratory fitness (CRF) and depression in adults.

**Methods:**

A total of 52,611 individuals aged between 18-59 years old were evaluated for symptoms of depression and CRF. The presence of depressive symptoms was self-report through the Patient Health Questionnaire (PHQ-9), and the CRF was predicted from a non-exercise equation. The association between CRF and the presence of depression was determined by crude and multivariable-adjusted logistic regressions.

**Results:**

The associations were identified between symptoms of depression and CRF in both unadjusted and adjusted models. After adjusting for age categories, sex, body mass index categories, educational level, marital status, smoking, and alcohol use, the individuals with moderate CRF had 18% lower odds of depression (OR: 0.82, CI 95%: 0.71 – 0.95) compared to individuals with low CRF.

**Conclusion:**

Depressive symptoms are inversely related to CRF levels in adults.

## 1 INTRODUCTION

Major Depressive Disorder (MDD) is a mental disorder characterized by profound dysregulation of mood and affect and loss of pleasure [1]. MDD is a common and debilitating disease that disturbs people from adolescence to old age [2-4], and around 264 million people have already been affected [5]. Furthermore, this mental disorder becomes increasingly recurrent in the presence of a new episode of major depression [6]. Given the above, the chronic and treatment-refractory courses still are a major challenge in the treatment of MDD [7]. According to World Health Organization (WHO), it is estimated that by 2030 MDD will be the most prevalent disease in the world [8]. In this scenario, the MDD imposes a vital cost to the economy [9], mainly affecting low- and middle-income countries [10, 11].

Some observational studies have shown a bidirectional association between MDD and cardiometabolic diseases as heart failure and coronary artery disease [12-14]. Thus, the risk of mortality is most significant in patients with treatment-resistant depression and insufficiently treated [15]. Besides, MDD patients have lower cardiorespiratory fitness (CRF) than the general population [16], which is associated with cardiovascular disease and risk of death [17]. In this context, low CRF in depressed patients is a critical risk factor due to its relationship with cardiovascular disease and premature mortality [18]. Thus, the improvement of CRF has been strongly recommended for these patients [19].

CRF consists of the body's ability to absorb, transport, and use oxygen during physical activity and exercise [20]. A meta-analysis showed that the severity of depressive symptoms is inversely related to CRF [21]. In turn, high CRF levels protect against future depressive episodes, regardless of factors, such as age, smoke, alcohol consumption, and other factors [22, 23]. Therefore, knowledge of CRF in this population would have important implications for health. However, CRF population data are scarce, probably due to operational problems and costs related to gold standard tests. Particularly in economically poorer countries, the lack of these data may represent a lack of information about health data.

Given this scenario, the CRF could be assessed by prediction models low cost and easy to apply. Given this scenario, the CRF could be predicted by prediction models, low cost, and easy to apply. In the literature, a predictive model of CRF uses multiple regression equations [24]. This predictive model has shown good predictive ability and accuracy of CRF [24]. To our knowledge, no report exists on this relationship between CRF and depression in population-based studies in low-income countries. At the same time, the literature has already shown a relationship between CRF and depression [21-23]. A broader understanding of CRF may provide strong evidence of depressive symptoms and significant implications for treating the condition, mainly in low-income and middle-income countries, where healthcare resources are limited [10]. Therefore, this study assessed the relationship between CRF and depression in adults.

## 2 METHODS

All the experimental procedures were explained to the volunteers, who then gave their informed consent. The study was approved by the National Research Ethics Committee (protocol number #328.159). It is an observational cross-sectional study where the participants completed individual questionnaires applied by interviewers trained by the Brazilian Institute of Geography and Statistics (IBGE) and the Ministry of Health. This survey was carried out throughout the national territory and the results may be seen on the IBGE website (http://www.ibge.gov.br/home/estatistica/populacao/pns/2013_vol3/default_microdados.shtm).

MDD was assessed through the Patient Health Questionnaire (PHQ-9) [25]. The PHQ-9 consists of nine questions to assess the frequency of depressive symptoms in the last two weeks. The frequency of each symptom in the last two weeks is evaluated on a 4-point Likert scale from 0 to 3, corresponding to the answers “Not at all,” “Several days,” “More than half the days,” and “Nearly every day,” respectively. As a diagnostic measure for MDD must be considered if ≥ 5 of the 9 symptoms criteria have been present at least “more than half the days” in the past 2 weeks, and if one of the symptoms is “depressed mood” or “anhedonia” or the criteria of “thoughts that you would be better off dead, or of hurting yourself in some way?” is present at all [25]. The PHQ-9 has been translated and validated in the Portuguese language [26]. In the epidemiological context of primary health care, the PHQ-9 may favor the early detection of depression. Those who showed less than two symptoms or negative responses for “depressive mood” and “lack of interest or pleasure” were considered without depression [25].

The CRF was estimated by Wier *et al*. [27]. For an estimate of the CRF, the following measures are needed: physical activity level, age, body mass index (BMI), and sex. Age, body mass index (BMI), and gender data were taken from the individual questionnaires IBGE website. The physical activity level was extracted from a valid and reliable questionnaire [28]. This questionnaire consists of questions related to domains of leisure, transportation, occupational, and household. The questionnaire uses two subject classification systems: “sufficiently active during leisure time” or “inactive in four domains of physical activity (leisure, work, transportation, and housework).” Individuals who informed the following criteria in the interview were classified as “sufficiently active during leisure time”: a) practicing physical exercise or sport of moderate intensity for at least 30 minutes a day on five; b) or more days a week or physical exercise or sport of vigorous intensity for at least 20 minutes daily on at least three days a week. Individuals classified as “inactive in four domains of physical activity” should inform the following in the telephone interview: a) they do not exercise or do sports at least one day a week; b) “do not walk frequently” and “do not often carry heavy loads” in their work (or have not worked in the last three months); c); are not responsible for the “heavy cleaning” of their homes; and d) do not commute from home to work on foot or by bicycle. Physical activity scores were developed applying the Physical Activity Rating (PA-R) scale by attributing a value that ranged from 0 to 10, which corresponded to the type, volume, and intensity of the physical activity reported [29].

Equation **1** is as follows:

VO_2_max=57.402+[1.396*(PA-R)]-[0.372*(age in years)]-[0.683*(BMI)]+[8.596*(sex; 0=female, 1=male)].

The predicted values were categorized into quartiles categorized by sex and age groups, with the extremes being classified as lower and higher fitness and 2^nd^ and 3^rd^ quartiles as intermediate.

### 2.1 Statistical Analysis

All calculations were performed using the statistical package Stata version 12.1 (Stata Corp, College Station, USA). Population characteristics are described by sex, age, physical activity level, marital status, educational level, current alcohol use, current smoker, depression status, and CRF groups. The Chi-square test was performed to assess the differences between variables (sex, age, physical activity level, marital status, educational level, current alcohol use, and current smoker) across depression status and CRF categories. Sampling weights and complex samples were considered using the “svy” command. All descriptive values were expressed as mean, standard deviation (SD), and absolute (*i.e*., number), and relative (*e.g*., percentage) values. The association between CRF and the presence of depression was determined by crude and multivariable-adjusted logistic regressions. Model 1 was the unadjusted model. Model 2 was adjusted for age, sex, excess body weight (BMI ≥25 kg.m-2), marital status, educational level, current alcohol use, and current smoking use. Odds ratios (OR) and confidence intervals (95% CI) were estimated for two models. The level of significance was set at 0.05.

## 3 RESULTS

Population characteristics are shown in Table **1**. The difference between the presence and absence of depression symptoms was significant for all variables. Depression symptoms were observed in 13.2% of the population.

The average age was 37.2±11.4 years old, and BMI was found 26.4±5.1 kg/m^2^ values. As for the physical activity level, 42.6% were classified as inactive, 44.9% as moderately active, and 12.4% as intensely active. In general, 59.9% were classified as excess body weight and 40.1% with normal body weight status. Only 13.7% had an educational level of ≥ 12 years, 36.4% between 9 and 11 years, and 49.9% less than eight years. Regarding marital status, 38.3% were married, and 61.7% were non-married. As for the current alcohol use, 45.9% consumed alcoholic beverages, and 54.1% did not drink alcoholic beverages. Regarding smokers, 15.4% were smokers, and 84.6% non-smokers.

The average estimated CRF was 32.5 ml.kg^-1^.min^-1^(SD 8.8). Moreover, the CRF categories of men and women according to quartiles are shown in Table **2**.

The association was observed in depression across different CRF categories in both models (*i.e*., unadjusted and adjusted, respectively) (Table **3**). Subjects with moderate CRF had 17% and 18% (models 1 and 2, respectively) lower odds of depression than individuals with low CRF. Besides, the individuals with high CRF had 29% and 28% (respectively unadjusted and adjusted) lower odds of depression than individuals with low CRF.

## 4 DISCUSSION

The study aimed to investigate the association between CRF and depression in adults. Our results confirmed that higher levels of CRF (*e.g*., moderate and high CRF, respectively) were associated with lower depressive symptoms, suggesting a beneficial effect of CRF on MDD. These results follow previous studies [30-32].

For instance, previous studies showed a higher prevalence of depression in women than men [25], which was demonstrated in our research. Furthermore, our findings indicated a higher prevalence of MDD between 18-29 years and 30-39 years. In this sense, a previous study suggested that the prevalence of mental disorders to age 32, including major depression, which was approximately doubled in prospective compared to retrospective data [31]. A previous study showed that a high prevalence occurs in the second and third decades of life across several countries (median between 18.8 and 31.9 years) [32]. In turn, the prevalence is lower from the fifth decade of life [32], following our findings (*i.e*., 19.3% at ages 50-59 years).

Concerning alcohol consumption, our results showed a two-fold higher prevalence of consumers of alcoholic beverages (68.8%) compared to non-consumers (31.2%). Based on this, the current state of the literature suggests that increasing alcohol involvement increases the risk of MDD in approximately 2.-folds [33]. In addition, the prevalence of smokers presenting depression symptoms was higher (21.1%) than the non-smokers (14.5%). The literature seems to indicate a relationship between both (smoke and depression); however, no causal relationship could be established [34]. In this sense, the relationship between smoke and depression may result from shared risk factors [34]. For bodyweight status, our findings demonstrated that excess body weight individuals have a higher prevalence of MDD (81.3%) compared to adults with normal weight (18.7%). Previous studies support the association between mood disorders and obesity [35]; however, the causal relationship between obesity and MDD remained unclear. In turn, the treatment of one condition (*i.e*., obesity or MDD) appears to improve the course of the other disease. In terms of marital status, our findings are in line with a previous study that showed that non-married people (*i.e*., single, widowed, separated, and divorced) have the highest hazard ratio for experienced major depression than those married [36]. Thus, a significant interplay exists between major depressive disorder and marital status. This risk is higher at the moment of marital disruption and during the divorced or separated marital status [36]. The last factor used as a covariate in this study is the educational level, which seems to be a mediator considering that lower educational level is associated with depression (effect size = 0.28) when compared to higher educational level (effect size = 0.05) [37].

As for the level of physical activity, our findings showed that only 12.4% of the population was categorized as intense activity, and only 3.1% of this category were depressed. In this scenario, although this investigation is a cross-sectional study, our findings suggest an association between the high level of physical activity and symptoms of depression. Corroborating, a meta-analysis demonstrated that people with higher physical activity levels had lower odds of developing depression (adjusted OR= 0.83) [38]. Another recent study showed the importance of physical activity level on the risk of depression development [39]. In general, this research reported that the duration of 40 min per day of physical activity (or ≥ 300 min per week) was associated with a significantly lower risk of depression than those who exercise below 150 min per week [39].

Given the above, it is suggested that a high level of physical activity promotes an increase in CRF [40]. It has increased interest in investigating an association between CRF and symptoms of depression [23, 41]. For instance, the study developed by Shigdel and colleagues [42], using a cohort study from Norwegian, showed an association between moderate and high CRF levels with lower levels of depression among middle-aged and older adults individuals in cross-sectional and longitudinal designs. A prospective study conducted by an average of 12 years of follow-up showed that men with moderate and high CRF have 31% and 49%, respectively, lower risk of developing depressive symptoms than men with low CRF (OR = 0.69 for moderate CRF; OR = 0.49 for high CRF). For women, moderate CRF has a 44% lower risk of developing depressive symptoms, and high CRF has a 54% lower risk of developing depression [23]. Therefore, our findings suggest a lower risk of developing depressive symptoms with moderate and higher CRF levels in men and women regardless of age. The mechanisms that lead to the association between CRF and depression have not yet been fully elucidated and remained inconclusive.

### 4.1 Strength and Limitations

Some studies [23, 41, 42] have investigated the association between CRF and depression in several populations, but little is known about this relationship in a populational-based study of low and middle-income countries. This research used a representative sample of middle-income country adults found evidence that CRF was associated with depression. In turn, there is not enough data to determine how much of the depressive symptoms burden may be due to low CRF levels. However, these findings could have implications for developing public policies using physical activity programs to increase CRF with consequences for mental health. Future studies should investigate the potential bidirectionality of the association. Furthermore, it would be helpful to investigate the mechanisms underlying the relationship between CRF and depression.

Our study presents some limitations. First, the study design was observational cross-sectional. Thus, it is not possible to detect a causal relationship between the variables. Second, the non-exercise equation to estimate CRF instead of the gold standard procedure [43]. Nevertheless, the use of the standard gold test in population study is not feasible. In addition, the use of non-exercise equations has been shown as an efficient alternative [43-45].

## CONCLUSION

In conclusion, depression was inversely associated with moderate-high CRF levels through a non-exercise model in adults from 18 to 59 years old. This finding suggests that a practical, viable, and low-cost CRF measurement could be applied in epidemiological studies of high financial costs.

## Figures and Tables

**Table 1:** Demographic, behavioral, and health characteristics of sample (n=52611).

-	Depression* **(n=6931)**	**No Depression** **(n=45680)**	**p**
**Sex**	-	-	-
Men	2932 (42.3%)	20599 (45.1%)	-
Women	3999 (57.7%)	25081 (54.9%)	<0.001
**Age Categories (Years)**	-	-	-
18-29	1935 (27.9%)	13550 (29.7%)	-
30-39	2009 (29.0%)	13299 (29.1%)	-
40-49	1647 (23.8%)	10569 (23.1%)	-
50-59	1340 (19.3%)	8262 (18.1%)	0.01
**Physical Activity Score (0-10)**	-	-	-
Inactive/Low Level (0-2)	1601(23.1%)	20830 (45.6%)	-
Moderate Activity (3)	5117 (73.8%)	18529 (40.6%)	-
Intense Activity (≥4)	213 (3.1%)	6321 (13.8%)	<0.001
**Body Weight Status**	-	-	-
Normal	1293 (18.7%)	19794 (43.3%)	-
Excess body weight	5638 (81.3%)	25886 (56.7%)	<0.001
**Marital Status**	-	-	-
Married	2332 (33.7%)	17793 (38.9%)	-
Non-married	4599 (66.3%)	27887 (61.1%)	<0.001
**Educational Level (Years)**	-	-	-
0-8	3620 (52.2%)	22639 (49.6%)	-
9-11	2455 (35.4%)	16696 (36.5%)	-
≥ 12	856 (12.4%)	6345 (13.9%)	<0.001
**Current Alcohol Use**	-	-	-
Yes	4771 (68.8%)	19385 (42.4%)	-
No	2160 (31.2%)	26295 (57.6%)	<0.001
**Current Smoker**	-	-	-
Yes	1463 (21.1%)	6618 (14.5%)	-
No	5468 (78.9%)	39062 (85.5%)	<0.001

**Table 2:** Cardiorespiratory Fitness classification of men (n = 23531) and women (n = 29080) in according to quartiles (n=23531).

-	**Men**	-
**Age Categories**	**Low (1st)**	**High (4th)**
18-29	≤ 42,11	≥51,43
30-39	≤ 35,48	≥44,01
40-49	≤ 30,81	≥38,55
50-59	≤ 26,93	≥33,99
**Women**		
18-29	≤ 28,88	≥36,11
30-39	≤ 23,36	≥30,54
40-49	≤ 18,73	≥25,75
50-59	≤ 14,40	≥21,47

**Table 3:** Unadjusted and Adjusted odds of depression (n=52611).

-	**Depression**			** Model 1^a^** **OR **	**IC95%**	**Model 2^b^** OR*	**IC95%**
	**Yes**	**No**					
**CRF (Quartiles)**	-			-	-	-	-
Low (1st)	4380		11871	1 (referent)	-	1 (referent)	-
Moderate (2nd-3rd)	1639		22591^c^	0.83^*^	0.72-0.95	0.82^*^	0.71-0.95
High (4th)	912		11218^c^	0.71^**^	0.61-0.84	0.72^**^	0.59-0.87

^a^unadjusted. ^b^adjusted by age categories, sex, excess body weight (≥25 kg.m^-2^), marital status, educational level, current alcohol, current smoker. ^c^p for trend <0.001. ^*^p=0.01. ^**^p<0.001.

## Data Availability

Not applicable.

## LIST OF ABBREVIATIONS

CRF: = Cardiorespiratory fitness
PHQ-9: = Patient Health Questionnaire-9
OR: = Odds Ratio
CI: = Confidence Interval
MDD: = Major Depressive Disorder
WHO: = World Health Organization
IBGE: = Brazilian Institute of Geography and Statistics
BMI: = Body Mass Index
PA-R: = Physical Activity Rating
VO_2_max: = Maximal oxygen consumption
SD: = Standard Deviation

## References

[1] Association A.P. (2013). Diagnostic and statistical manual of mental disorders (DSM-5®)..

[2] McKeever A., Agius M., Mohr P. (2017). A review of the epidemiology of major depressive disorder and of its consequences for Society and the individual.. Psychiatr. Danub..

[3] Mura G., Carta M.G. (2013). Physical activity in depressed elderly. A systematic review.. Clin. Pract. Epidemiol. Ment. Health.

[4] Bukh J.D., Bock C., Vinberg M., Gether U., Kessing L.V. (2011). Differences between early and late onset adult depression.. Clin. Pract. Epidemiol. Ment. Health.

[5] Roberts NL, Mountjoy-Venning WC, Anjomshoa M, Banoub JAM, Yasin YJJL, GBD 2017 disease and injury incidence and prevalence collaborators (2019). Global, regional, and national incidence, prevalence, and years lived with disability for 354 diseases and injuries for 195 countries and territories, 1990-2017: A systematic analysis for the Global Burden of Disease Study (vol 392, pg 1789, 2018).

[6] Spijker J., de Graaf R., Bijl R.V., Beekman A.T., Ormel J., Nolen W.A. (2002). Duration of major depressive episodes in the general population: Results from The Netherlands Mental Health Survey and Incidence Study (NEMESIS).. Br. J. Psychiatry.

[7] Bauer M., Rush A.J., Ricken R., Pilhatsch M., Adli M. (2019). Algorithms for treatment of major depressive disorder: Efficacy and cost-effectiveness.. Pharmacopsychiatry.

[8] Organization W.H. (2008). The global burden of disease: 2004 update..

[9] Carta M.G., Preti A., Portoghese I., Pisanu E., Moro D., Pintus M., Pintus E., Perra A., D’Oca S., Atzeni M., Campagna M., Pascolo E.F., Sancassiani F., Finco G., D’Aloja E., Grassi L. (2017). Risk for depression, burnout and low quality of life among personnel of a university hospital in Italy is a consequence of the impact one economic crisis in the welfare system?. Clin. Pract. Epidemiol. Ment. Health.

[10] Malhi G.S., Mann J.J. (2018). Depression.. Lancet.

[11] Ojagbemi A., Gureje O. (2019). Typology of social network structures and late-life depression in low- and middle-income countries.. Clin. Pract. Epidemiol. Ment. Health.

[12] Tang B., Yuan S., Xiong Y., He Q., Larsson S.C. (2020). Major depressive disorder and cardiometabolic diseases: A bidirectional Mendelian randomisation study.. Diabetologia.

[13] de Ornelas Maia A.C., Braga Ade.A., Paes F. (2013). Comorbidity of depression and anxiety: Association with poor quality of life in type 1 and 2 diabetic patients.. Clin. Pract. Epidemiol. Ment. Health.

[14] Compare A., Germani E., Proietti R., Janeway D. (2011). Clinical psychology and cardiovascular disease: An up-to-date clinical practice review for assessment and treatment of anxiety and depression.. Clin. Pract. Epidemiol. Ment. Health.

[15] Scherrer J.F., Chrusciel T., Garfield L.D. (2012). Treatment-resistant and insufficiently treated depression and all-cause mortality following myocardial infarction.. Br. J. Psychiatry.

[16] Boettger S., Wetzig F., Puta C. (2009). Physical fitness and heart rate recovery are decreased in major depressive disorder.. Psychosom. Med..

[17] Kodama S., Saito K., Tanaka S., Maki M., Yachi Y., Asumi M., Sugawara A., Totsuka K., Shimano H., Ohashi Y., Yamada N., Sone H. (2009). Cardiorespiratory fitness as a quantitative predictor of all-cause mortality and cardiovascular events in healthy men and women: a meta-analysis.. JAMA.

[18] Vancampfort D., Rosenbaum S., Schuch F. (2017). Cardiorespiratory fitness in severe mental illness: A systematic review and meta-analysis.. Sports Med..

[19] Stubbs B., Rosenbaum S., Vancampfort D., Ward P.B., Schuch F.B. (2016). Exercise improves cardiorespiratory fitness in people with depression: A meta-analysis of randomized control trials.. J. Affect. Disord..

[20] Lamoureux N.R., Fitzgerald J.S., Norton K.I., Sabato T., Tremblay M.S., Tomkinson G.R. (2019). Temporal trends in the cardiorespiratory fitness of 2,525,827 adults between 1967 and 2016: A systematic review.. Sports Med..

[21] Papasavvas T., Bonow R.O., Alhashemi M., Micklewright D. (2016). Depression symptom severity and cardiorespiratory fitness in healthy and depressed adults: A systematic review and meta-analysis.. Sports Med..

[22] Dishman R.K., Sui X., Church T.S., Hand G.A., Trivedi M.H., Blair S.N. (2012). Decline in cardiorespiratory fitness and odds of incident depression.. Am. J. Prev. Med..

[23] Sui X., Laditka J.N., Church T.S. (2009). Prospective study of cardiorespiratory fitness and depressive symptoms in women and men.. J. Psychiatr. Res..

[24] Wang Y., Chen S., Lavie C.J., Zhang J., Sui X. (2019). An overview of Non-exercise estimated cardiorespiratory fitness: Estimation equations, cross-validation and application.. J Sci Sport.

[25] Kroenke K., Spitzer R.L., Williams J.B. (2001). The PHQ-9: validity of a brief depression severity measure.. J. Gen. Intern. Med..

[26] de Lima Osório F., Vilela Mendes A., Crippa J.A., Loureiro S.R. (2009). Study of the discriminative validity of the PHQ-9 and PHQ-2 in a sample of Brazilian women in the context of primary health care.. Perspect. Psychiatr. Care.

[27] Wier L.T., Jackson A.S., Ayers G.W., Arenare B. (2006). Nonexercise models for estimating VO2max with waist girth, percent fat, or BMI.. Med. Sci. Sports Exerc..

[28] Monteiro C.A., Florindo A.A., Claro R.M., Moura E.C. (2008). Validity of indicators of physical activity and sedentary lifestyle obtained by survey phone.. Rev Public Health.

[29] Neto GM, Pedreiro R, Oliveira A, Machado S, Vieira L, Neto SM (2019). Estimate of cardiorespiratory fitness of the Brazilian population of 20 to 59 years old: approach through model without exercise with self-reported variables.

[30] Sancassiani F., Machado S., Preti A. (2018). Physical activity, exercise and sport programs as effective therapeutic tools in psychosocial rehabilitation.. Clin. Pract. Epidemiol. Ment. Health.

[31] Moffitt T.E., Caspi A., Taylor A. (2010). How common are common mental disorders? Evidence that lifetime prevalence rates are doubled by prospective *versus* retrospective ascertainment.. Psychol. Med..

[32] Kessler R.C., Bromet E.J. (2013). The epidemiology of depression across cultures.. Annu. Rev. Public Health.

[33] Boden J.M., Fergusson D.M. (2011). Alcohol and depression.. Addiction.

[34] Fluharty M., Taylor A.E., Grabski M., Munafò M.R. (2017). The association of cigarette smoking with depression and anxiety: A systematic review.. Nicotine Tob. Res..

[35] Jantaratnotai N., Mosikanon K., Lee Y., McIntyre R.S. (2017). The interface of depression and obesity.. Obes. Res. Clin. Pract..

[36] Bulloch A.G., Williams J.V., Lavorato D.H., Patten S.B. (2009). The relationship between major depression and marital disruption is bidirectional.. Depress. Anxiety.

[37] Bjelland I., Krokstad S., Mykletun A., Dahl A.A., Tell G.S., Tambs K. (2008). Does a higher educational level protect against anxiety and depression? The HUNT study.. Soc. Sci. Med..

[38] Schuch F.B., Vancampfort D., Firth J. (2018). Physical activity and incident depression: A meta-analysis of prospective cohort studies.. Am. J. Psychiatry.

[39] Hallgren M., Nguyen T.T., Lundin A. (2019). Prospective associations between physical activity and clinician diagnosed major depressive disorder in adults: A 13-year cohort study.. Prev. Med..

[40] Savi D., Di Paolo M., Simmonds N. (2015). Relationship between daily physical activity and aerobic fitness in adults with cystic fibrosis.. BMC Pulm. Med..

[41] Suija K., Timonen M., Suviola M., Jokelainen J., Järvelin M.R., Tammelin T. (2013). The association between physical fitness and depressive symptoms among young adults: results of the Northern Finland 1966 birth cohort study.. BMC Public Health.

[42] Shigdel R., Stubbs B., Sui X., Ernstsen L. (2019). Cross-sectional and longitudinal association of non-exercise estimated cardiorespiratory fitness with depression and anxiety in the general population: The HUNT study.. J. Affect. Disord..

[43] Wang Y, Chen S, Lavie CJ, Zhang J (2019). Sui XJJoSiS, Exercise. An overview of Non-exercise estimated cardiorespiratory fitness: estimation equations, cross-validation and application.

[44] Neto MGA, Oliveira AJ, Pedreiro R (2017). Prediction of cardiorespiratory fitness from self-reported data in elderly..

[45] Maranhao Neto G.A., Alves I., Lattari E. (2020). Association between type 2 diabetes and non-exercise estimated cardiorespiratory fitness among adults: evidences from a middle-income country.. Public Health.

